# Investigation of mechanical, physical and thermoacoustic properties of a novel light-weight dense wall panels made of bamboo *Phyllostachys Bambusides*

**DOI:** 10.1038/s41598-023-45515-3

**Published:** 2023-10-26

**Authors:** Parham Gholizadeh, Hamid Zarea Hosseinabadi, Dirk E. Hebel, Alireza Javadian

**Affiliations:** 1 Department of Architecture, Faculty of Arts and Architecture, University of Mazandaran, Babolsar, Iran; 2 Department of Wood and Paper Science and Technology, Faculty of Natural Resources, University of Tehran, Karaj, Iran; 3 Faculty of Architecture, Karlsruhe Institute of Technology, Karlsruhe, Germany

**Keywords:** Engineering, Composites

## Abstract

This study was conducted to evaluate the properties of lightweight sandwich panels made from low diameter bamboo particles, Phyllostachys Bambusides collected from Gilan province, Iran, as core layer, combined with thin wall bamboo strips as faces. The effects of three individual variables such as density of core layer (350–550 kg/m^3^), resin consumption in core layer (7.5–9.5%) and resin consumption in faces (175–275 g/m^2^) on some important physical, mechanical and thermos-acoustic properties of the panels were investigated. Response surface methodology was used to statistically analyse the results and optimization process. The average values for the mechanical properties of the sandwich panels were obtained as 17.16 MPa, 5669 MPa, 0.02 MPa, 17.60 MPa, 1.83 MPa, 0.03 MPa, and 913.3 MPA for modulus of rupture, modulus of elasticity, internal bonding, compression strength parallel to face grain, compression strength perpendicular to face grain, shear strength, and screw holding, respectively. Finally, thermal conductivity and noise reduction coefficient of the panels were respectively gained as 0.01 W/mk and 0.31. The results of technical and thermo- acoustic properties of the panels showed that the light weight sandwich panels from bamboo residues would be a suitable and sustainable alternative as an insulation material for sustainable and green construction.

## Introduction

It is clear as of today that alternative materials to wood is urgently needed to reduce logging from global forests and reduce the pressure on our forest reserves and to meet the rising demand for wood-based products^1,2^. According to estimates from the UN FAO, 10 million hectares of forest are destroyed annually^3^, to be more clear, forestry covered 31.6 percent of the planet’s surface in 1990; by 2022, this number was dropped to 31 percent^4,5^. Besides, with the improvement of living standards, the demand for environmentally friendly, lightweight, and sustainable structures have increased dramatically. Bamboo as a sustainable alternative to traditional building materials, including wood, has started to gain attention due to its fast growth rate and the short time to harvest, the variety of species, as well as the high yield, and the ability to reach a maximum height of 15–30 m in 2–4 months, and reaching the maximum strength in 3–5 years. According to life-cycle assessment (LCA) results, bamboo is included in the “factor 20”, which means that its impact on the environment is 20 times less compared to modern alternatives^6,7^. Simply put, the sustainability of bamboo-based building materials is governed by relatively fast harvesting, a more efficient rate of carbon sequestration compared to wood species, as well as low-energy requirement for processing, which creates minimal environmental impact^8^. Additionally, to make the material more environmentally friendly, bamboo waste can be used in the production of other products including bamboo-based building panels, which have been investigated in this research.

Furthermore, compared to wood species, bamboo has great mechanical characteristics, a shorter rotation, fast and convenient processing, low density, and is biodegradable, all of which have drawn increased attention^9–12^. However, the processing of bamboo is different from that of wood because of its heterogeneous structure, which mostly comprises of vascular bundles and parenchyma cells^13^. Due to its distinctive multi-level pore structures and densely packed fibres/lignin composite as elastic and robust wall material, bamboo, a work of art of nature, exhibits lightweight and great mechanical capabilities^14^. Bamboo plywood, laminated bamboo lumber, bamboo particleboard, and bamboo fibre reinforced polymer composites are some of the bamboo-based products that have been developed recently^15–21^. These products are widely used in a variety of industries, including construction, furniture and flooring^22^. For instance, with the use of an oriented bamboo fiber mat coated with Phenol Formaldehyde resin, Yu et al. created a unique bamboo scrimber. Additionally, the siliceous and waxy layers were taken out. As a result, the bamboo scrimber's modulus of rupture (MOR) reached 253 MPa, which was significantly higher than those of natural bamboo and conventional bamboo scrimber^23^. Verma and Chariar^24^ investigated that a comparative cost and mechanical properties analysis of LLBCs (layered laminated bamboo composites) with teak wood timber indicates that LLBCs could be used as building and general purposes material like, furniture, beam and column, etc., because there is possibility to increase the volume in any shape and in any direction by increasing number of layers. Sharma et al., carried out an investigation into the mechanical properties of bamboo scrimber and laminated bamboo composite to assess their potential for structural applications and to compare them with timber and engineered timber products. The observations showed that an advantage of laminated bamboo to timber and raw bamboo is its bending strength to density ratio namely specific strength. The results of the study indicate that both products; bamboo scrimber and laminated bamboo have properties that compare with or surpass that of timber^25^. In an experimental study, Fadlelmola et al., investigated the bamboo-concrete sandwich panel, steel–concrete sandwich panel and plain concrete panel under bending load. The failure modes were attributed to bamboo tensile yielding at the middle of the bottom face, followed by Aerated Lightweight Concrete shear cracks at the maximum shear point. The results show that using bamboo in sandwich panels highly increases the structural properties such as moment capacity, ductility and bending stiffness compared to non-reinforced concrete^26^.

Investigation of physical and mechanical properties of bamboo has an important role in its application in various structures. Structures made of bamboo should be designed and evaluated with respect to the bending, shear, tensile and compressive strength as well as stability in dynamic loading. Studies have been carried out by various researchers that have been summarized in previous section. The major difference in the works mentioned is the type of bamboo panel used and its properties^27,28^.

In terms of insulating properties of engineered composites, there are environmental issues that should be considered. A sound absorber consisting of synthetic fibers like glass wool, rock wool, or foam glass, which is still utilized in the building sector, is one of the causes that contribute to the global warming problem. Asdrubali discovered that although they are effective at isolating heat and sound, they have the biggest potential for contributing to global warming when compared to natural materials^29^. Research is now directed towards finding an alternative acoustic absorber made from natural materials. There are some works carried out on the effective role of bamboo structure on its thermoacoustic properties. Khair et al., presents the preliminary study on the effect of the hollow structure of bamboo to absorb sound energy. The result shows good sound absorption at high frequency above 3000 Hz with bamboo sample of 2 cm long and micro-holes of 0.4 mm in diameter. Improvement of absorption coefficient at lower frequency can be achieved by increasing the air gap at the back of the sample^30^.

The characteristics of sandwich panels made of other materials can also be mentioned. Haseli et al., studied the characteristics of lightweight value-added sandwich panels, block boards and wooden boards, made of palm waste trunks as the core layer along with thin Medium Density Fiberboard as the top layers. Based on the experimental results, palm wood-MDF sandwich panels with good thermal conductivity of about 0.14 W/mK and favourable sound absorption of up to 0.64 and noise reduction coefficients up to 0.15 can be used as heat and sound insulating materials^31^.

The present study is investigating the suitability of semi-lightweight bamboo sandwich panels made of three layers, where the core is a particleboard consist of low diameter bamboo particles combined by thin bamboo strips as faces for sound insulation. Our study explores the potential of bamboo sandwich panels with three layers. These panels consist of a core made from finely ground bamboo particles, surrounded by thin bamboo strips that help with sound insulation. We’ve come up with a new sandwich design using bamboo from Iran, which is an important type of bamboo in Asia called *Phyllostachys Bambusides*. We’ve thoroughly studied how this bamboo composite performs physically, mechanically, and in terms of sound insulation. Our findings show that this design works well for making internal walls that don't bear heavy loads but have good insulation. In summary, our research demonstrates how bamboo, especially the *Phyllostachys Bambusides* from Iran, can be used to create effective sandwich panels. These panels could be used to build sound-insulated internal walls, offering a sustainable and practical solution.

## Materials and methods

### Raw materials

Four-year-old bamboo (*Phyllostachys Bambusides*) stems were used in this study. supplied from Lialestan bamboo plant, Gilan, I. R. Iran. The collection of the bamboo samples was carried out according to the Convention on Biological Diversity. In this study, Methylene diphenyl diisocyanate (MDI) resins (solid content: 100%, viscosity: 300 cP, density: 1.27 g cm^3^) were provided from Karoon Petrochemical co, I. R. IRAN. Polyurethane adhesive (color: cream, pot life: 25 min., density: 1.2 g cm^3^, solid content: 100%) for face strips attachment was supplied by Mokarrar Industrial Group, I. R. Iran.

### Design of experiment

The bamboo strips and flakes were glued with PU and MDI resins according to the design of experiment (Table 1). Response Surface Methodology (RSM) under central composite design in Design Expert version DX13 computer software package was used to design the experiment and analyze the results. In RSM, errors are assumed to be random. The application of RSM for design optimization is to reduce the cost of expensive analysis methods and the numerical irregularities associated with them (such as CFD or finite element analysis). In RSM, convergence towards the optimal element is preferred because they reduce the effects of disorder factors. In this design, 3 variables (density, core layer resin, skin resin) were considered in 5 levels, and as a result, 15 laboratory panels were produced.

**Table 1:** Specified levels of variables.

Variables	− α	− 1	0	+ 1	+ α
Density (kg/m^3^)	350	400	450	500	550
Core layer resin (%)	7.5	8	8.5	9	9.5
Skin resin (g/m^2^)	175	200	225	250	275

To follow the partial factorial design, 15 laboratory lightweight sandwich panels (density of 600 ± 60 kg m^3^) were manufactured (Table 2).

**Table 2:** Design of experiments table.

Run	Factor A: core density	Factor B: resin (core)	Factor C: resin (face)
1	450	8.5	225
2	450	7.5	225
3	500	8	250
4	400	9	250
5	450	8.5	175
6	350	8.5	225
7	500	8	200
8	400	8	250
9	450	9.5	225
10	500	9	200
11	450	8.5	275
12	400	9	200
13	550	8.5	225
14	500	9	250
15	400	8	200

### Fabrication of panels

#### Strip preparation and treatment

Strip preparation has been carried out in Wood—based material laboratory, Department of Wood and Paper 

**Figure 1:**
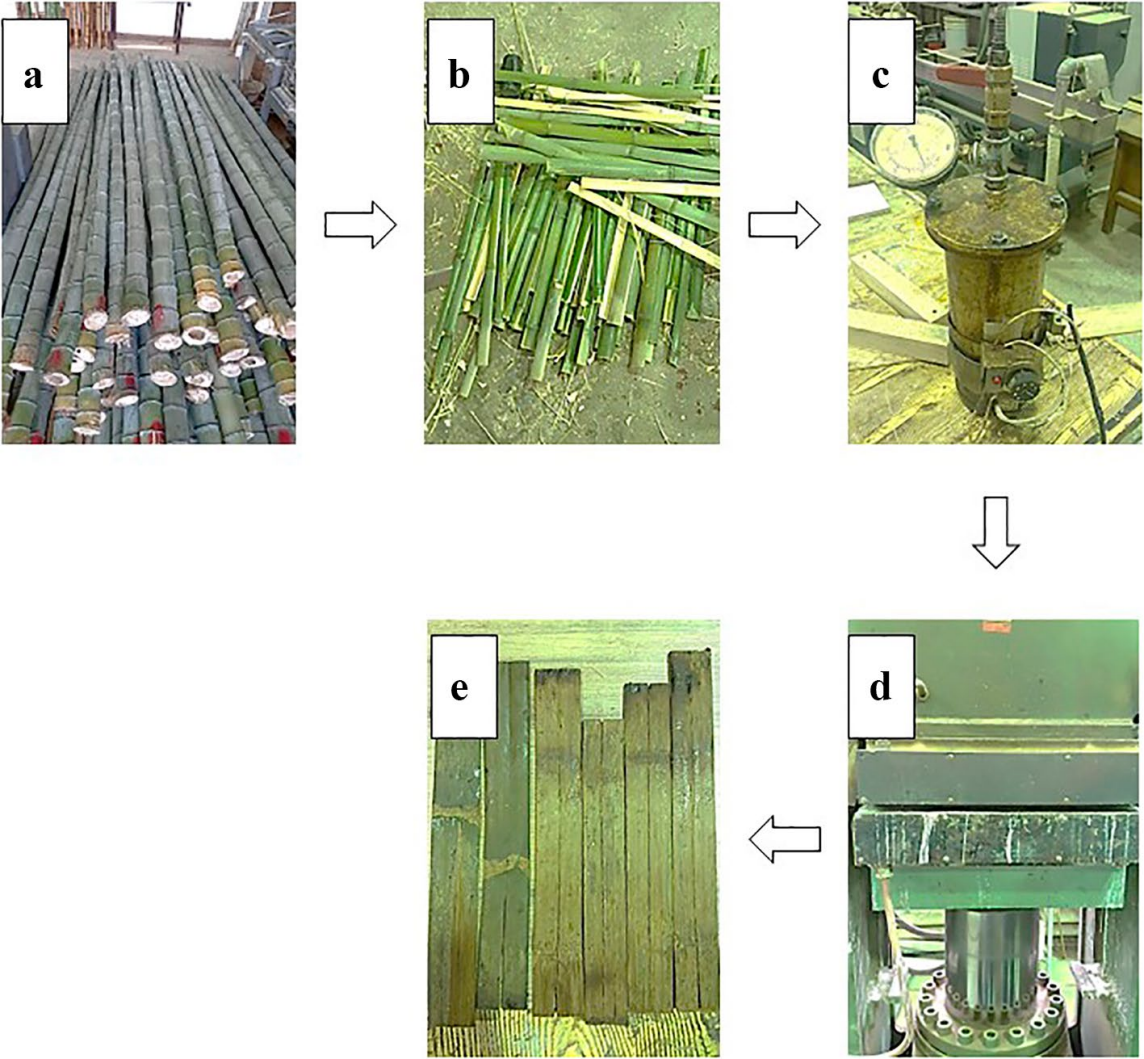
Strip preparation. (**a**) Bamboo stem, (**b**) bamboo strip, (**c**) strip steam treatment, (**d**) hot pressing of the treated strips, (**e**) flattened strips.

Science at University of Tehran. Strips were randomly extracted from the length of bamboo culms. This method was chosen to reduce the effect of uncontrollable factors in the final results of the tests, because the presence of nodes along the strip and the extraction location of the strips were not among the variable factors of this research. Bamboo stems (Fig. 1a) were cut into the pieces with around 50 cm were longitudinally cut into the suitable strips (Fig. 1b). For steam treatment, the strips were placed inside a laboratory cooking cylinder (Fig. 1c). Cooking temperature and steaming time inside the cylinder were 150 °C and 1 h, respectively. The pressing operation was performed immediately on the treated strips, in such a way that the strips were placed under a heat press with a temperature of 120 °C and a pressure of 20 kg/cm^2^ (Fig. 1d). The flattened strips were placed under heavy steel plates for 24 h and prepared for use in the assembly stage (Fig. 1e). To control the moisture content of the strips, they were kept in a conditioning chamber at 20 ± 3 °C and 65 ± 1 percent RH with constant temperature and humidity for a week.

#### Flake preparation

**Figure 2:**
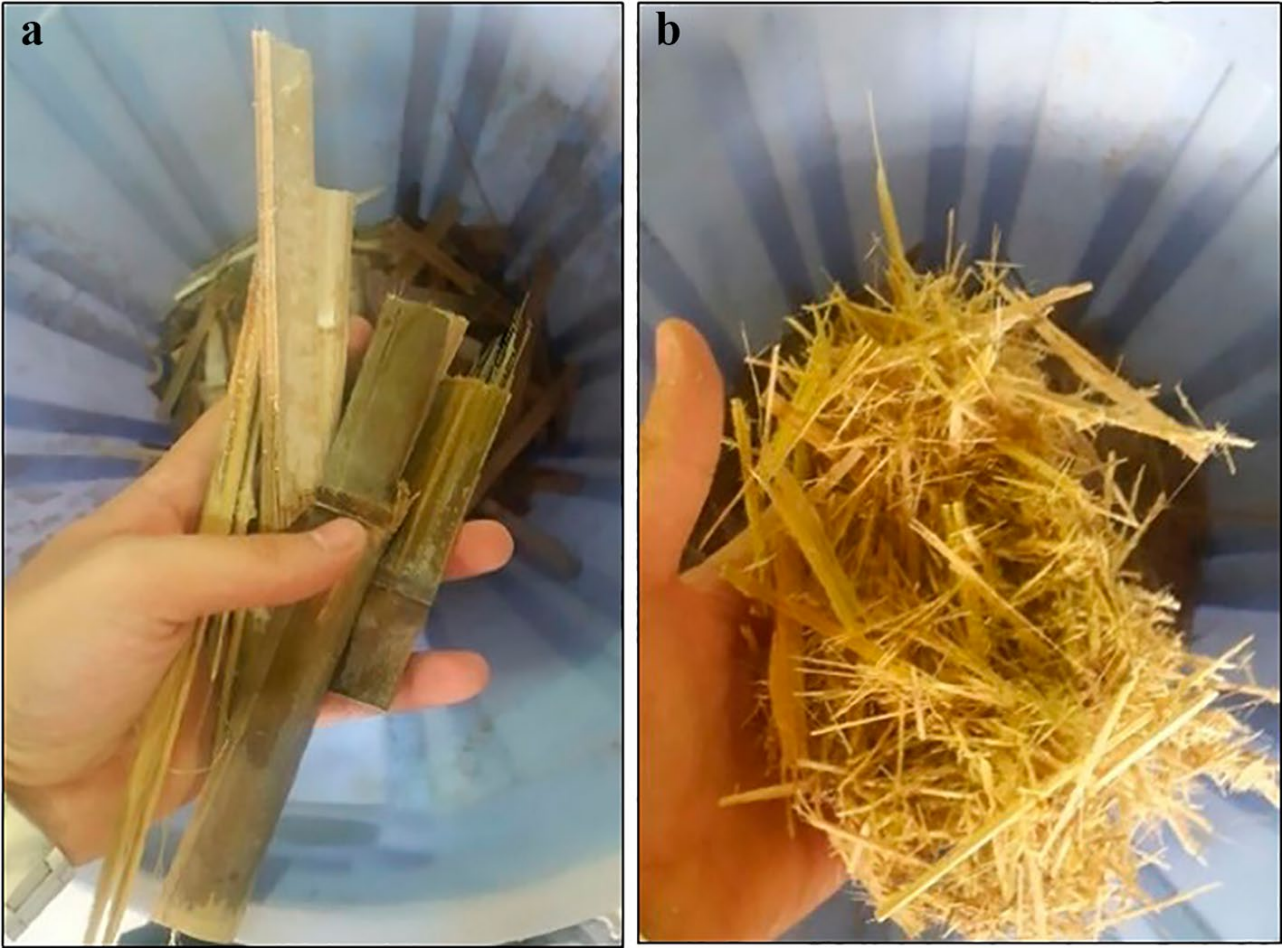
Raw material preparation for core layer (**a**) bamboo chips, (**b**) bamboo flake.

Bamboo flakes were used to make the middle layer of the sandwich panels. After delivering low quality bamboo strips and scraps to the laboratory, the bamboo pieces were cut into the smaller ones(8 < x < 15) (Fig. 2a). Knife length, using a band saw. The short stems ring flaker (Pallmann, Germany) was used to convert the bamboo chips into the suitable flaker, in order to obtain the appropriate raw material for making the middle layer of the sandwich panels (Fig. 2b). The bamboo flake was placed in a laboratory tray dryer at 105 °C for 24 h to achieve a moisture content around 2%, then passed through a laboratory flat vibratory screen to separate acceptable sizes for 40 ≤ x ≤ 80 mm. Dried and screened flakes were stored in sealed plastic bags before manufacturing the core panel.

#### Light weight panel manufacturing

**Figure 3:**
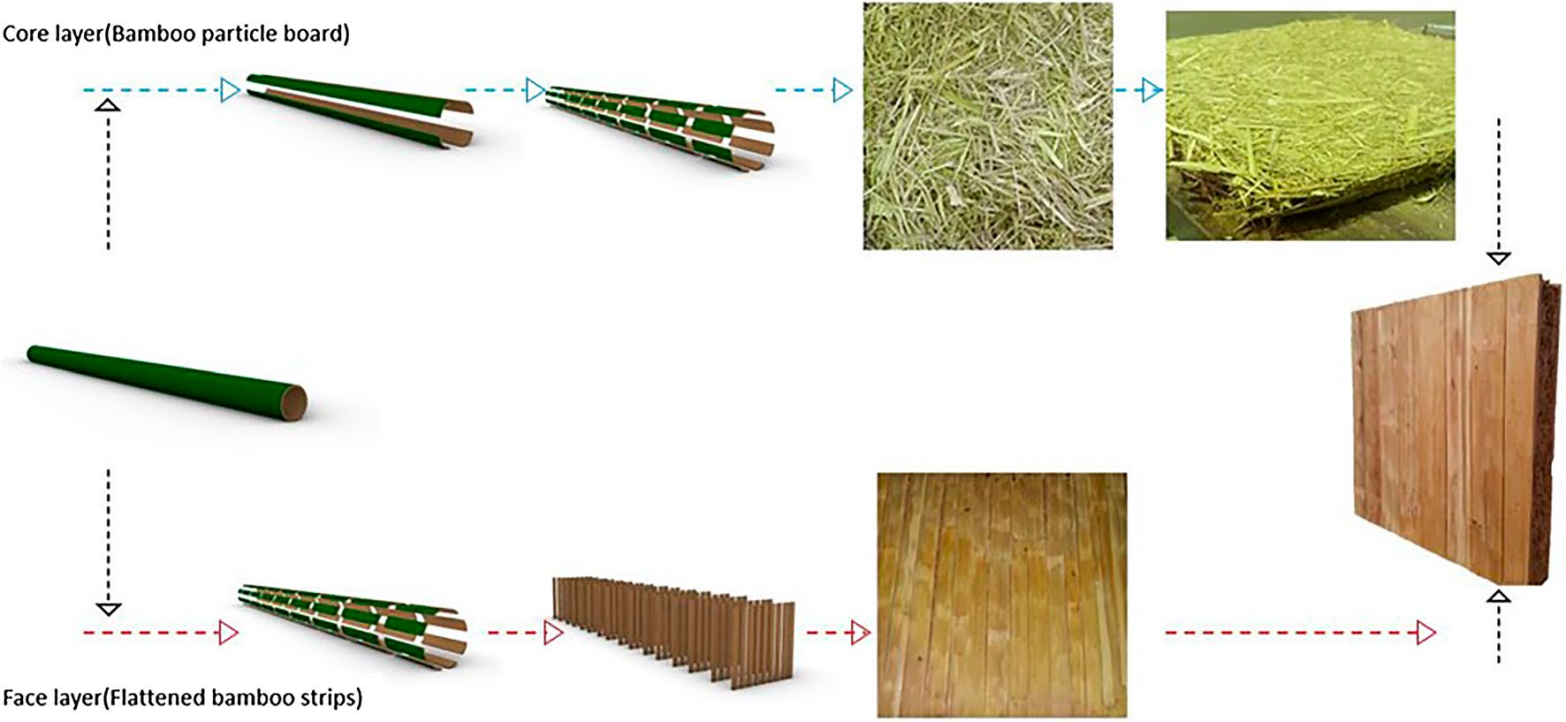
The panel production process in schematic form.

The schematic view of panel manufacturing is shown in Fig. 3. The panel specifications and parameters for making lightweight sandwich wall panel are presented in Table 3.

**Table 3:** Constant processing parameters in the production of panels.

Processing parameter	Value
PU content	Face 7.5–9.5%
MDI content	Core 175–275 g/m^2^
Board moisture content	6.6%
Board type	Sandwich panel
Target density	Panel 608 kg/m^3^
Dimensions	45 × 45 × 2.5 cm
Nominal thickness	2.5 cm
Pressing pressure	12 kg/cm^2^
Press closing time	4 mm/s
Pressing temperature (ºC)	100
Pressing time	45 min

**Figure 4:**
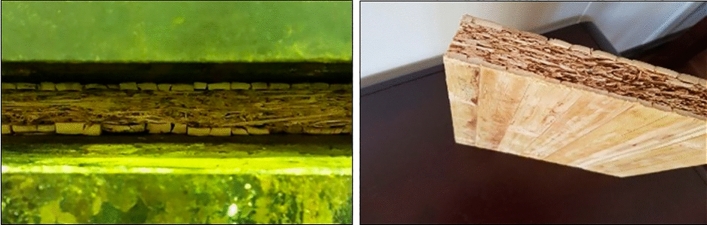
Three layers (core- two outer layers) of sandwich panel under the pressure.

Diluted adhesive was sprayed onto bamboo particles with coherent parameters using a laboratory rotational drum mixer having an internal spray nozzle. A mixture of bamboo chips and MDI resin was taken out of the gluing machine and manually formed into a 45 × 45 × 2.5 cm frame. At this stage, Burkle laboratory hot press was used. The press temperature was adjusted to 180 °C and a pressure of 24 kg/cm^2^ was applied for 8 min. Polyurethane (PUR) adhesive used for bamboo strips in outer layers. The press was set at a pressure of 12 kg/cm^2^, at a temperature of 100 °C, and the panels were pressed for 45 min. Due to the usage of polyurethane resin that does not require heat, we could use no heat for the final assemble, but to speed up the production of the samples, it was decided to use heat during the pressing (Fig. 4).

### Panel characterization

All treated panels were stored in a conditioning chamber for two weeks after cold stacking, at 20 ± 3 °C and 65 ± 1 percent RH, in line with (ASTM D1037^32^) until the panels acquired the standard moisture equilibrium content.

### Panel characterization

#### Physical and mechanical properties

Physical and mechanical tests along with thermos-acoustic properties were measured in accordance with DIN EN, ISO and ASTM standards (Table 4). Several standards were used to perform the tests for two reasons. Firstly, it was due to the limitation in the dimensions and number of samples, and the second reason was the availability of equipment in the laboratory, each of which was designed for a specific standard**.**

**Table 4:** Standard and dimension of the test specimen.

Test title	Related standard	Specimen’s dimension(mm)	References
Density	DIN-EN323	50 × 50 × 25	^37^
Moisture content	DIN-EN322	50 × 50 × 25	^38^
Internal bonding	DIN-EN319	50 × 50 × 25	^39^
Modulus of rupture	DIN-EN310	250 × 50 × 25	^40^
Modulus of elasticity	DIN-EN310	250 × 50 × 25	^40^
Compression strength parallel to face grain	DIN-EN789	75 × 25 × 25	^41^
Compression strength perpendicular to face grain	ISO 3132	50 × 50 × 25	^42^
Compression- shear strength	ASTM D143	50 × 50 × 25	^43^
Screw holding	ASTM D1761	75 × 75 × 25	^44^
Thermal conductivity	ASTM C177	200 × 200 × 25	^33^
Noise reduction coefficient	ASTM C423-09a	Diameter: 99–29 Thickness: 25	^34^

**Figure 5:**
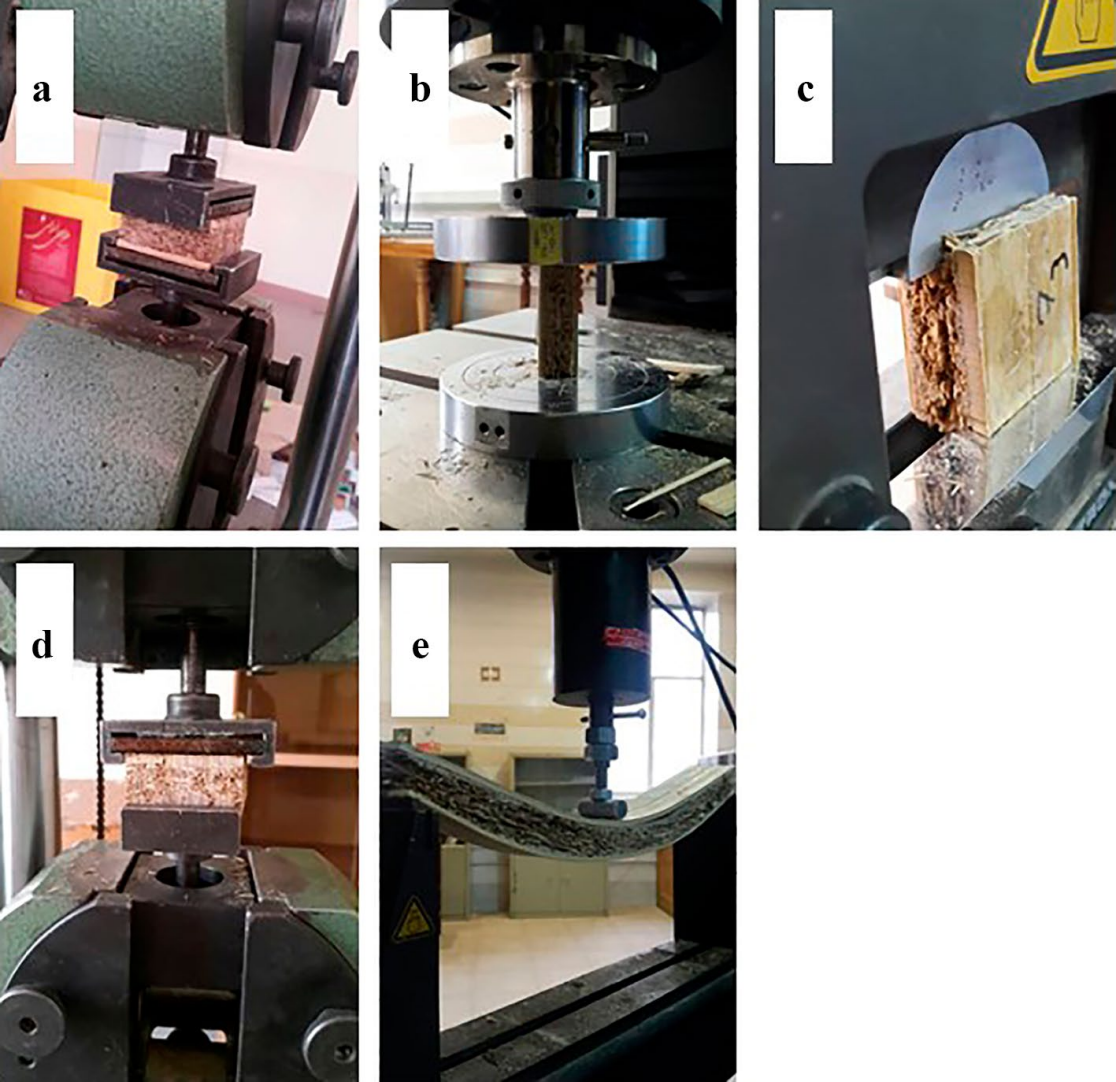
Mechanical characterization of the panels: (**a**) internal bonding, (**b**) compression strength parallel to face grain, (**c**) compression-shear strength, (**d**) screw holding, (**e**) three-point bending test.

Modulus of rupture, Internal bonding, screw holding and compression-shear tests were measured using universal testing machine (Wolpert, Germany), and modulus of elasticity, modulus of rupture and compression strength parallel and perpendicular to the face grain were measured using universal testing machine (Instron 4486) (Fig. 5a–e).

#### Thermo-accoustical testing

**Figure 6:**
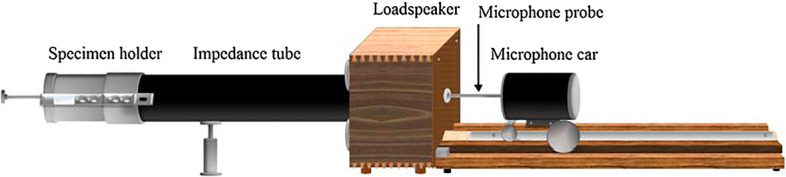
Noise reduction coefficient test.

Acoustic absorption coefficients and noise reduction coefficient (NRC) were used to determine acoustical properties of the panels. In this study, measurements were made in accordance with ASTM C423 Standard Method (ASTM C423^34^) using an impedance tube with two diameters as 99 mm and 29 mm (Fig. 6).

**Figure 7:**
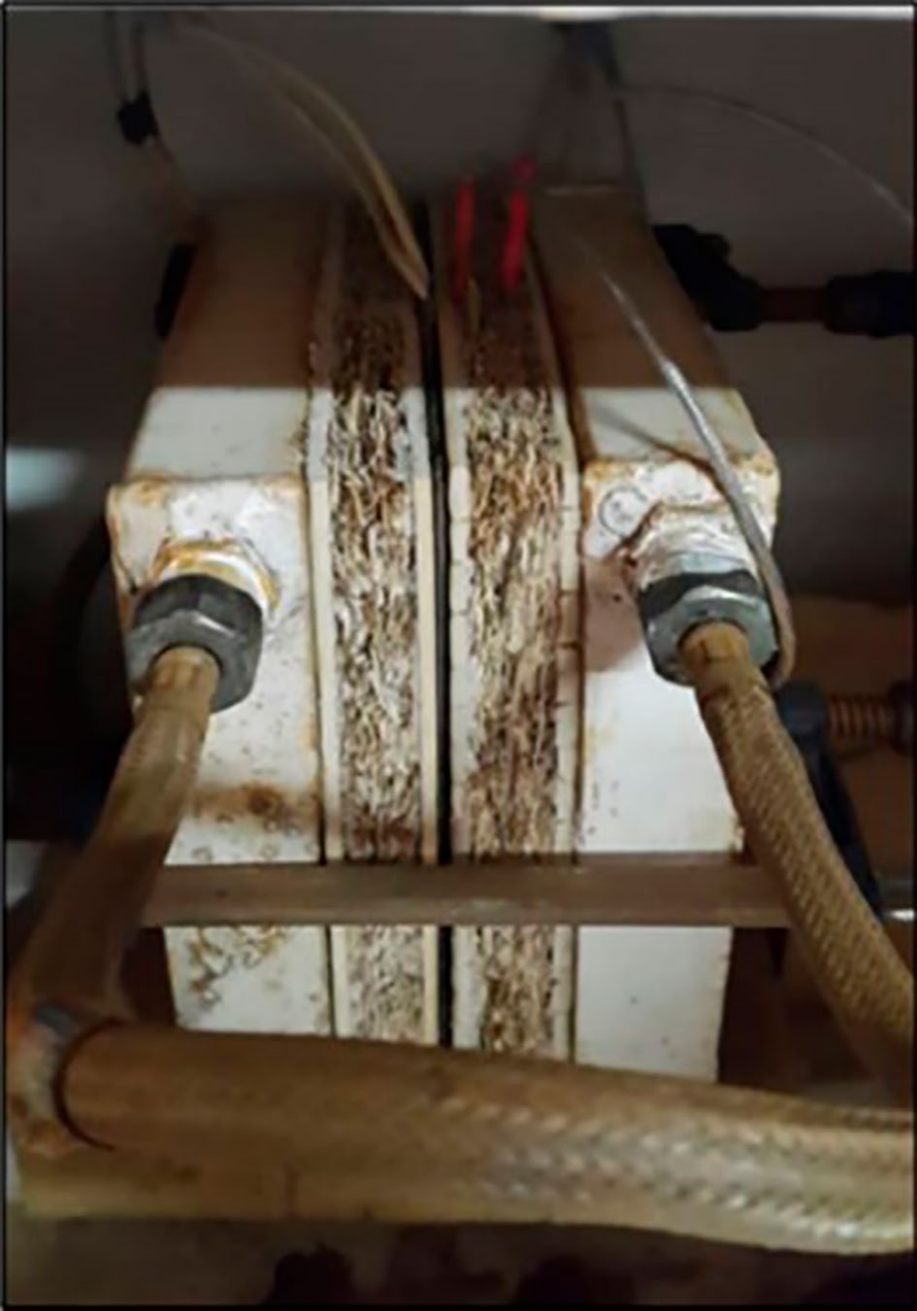
Thermal conductivity test.

The thermal conductivity of all panels (20 × 20 × 2 cm^3^) was measured at room temperature and normal pressure using the steadystate Bi-substrate technique (ASTM C177^33^) (Fig. 7).

### Numerical optimization

By numerical optimization in engineering sciences many phenomena according to their own instructions and conditions. However, a number of phenomena do not have the ability to have a suitable mathematical model due to the large number of controlling factors, unknown mechanism or computational complexity. In such cases, the use of experimental modeling methods works. The Response Surface Methodology (RSM) is considered as one of the experimental modeling methods^35^. RSM was originally developed to model experimental responses and then to model numerical experiments. In physical experiments, experimental errors can occur in a variety of ways, for example, estimating errors when the disorder or error is due to a wrong convergence (e.g., the operator is drowsy or tired or the test materials are heterogeneous) or to define a continuous physical phenomenon in a discrete way if such a thing cannot be done in reality. In RSM, the errors are assumed to be random. The application of RSM to design optimization is to reduce the cost of analytics methods and their associated numerical irregularities (such as CFD or finite element analysis). Also, convergence towards the element is optimal because they reduce the effects of disorder factors.

### Ethics statement

Four-year-old bamboo (*Phyllostachys Bambusides*) stems were used in this study. supplied from Lialestan bamboo plant, Gilan, Iran. The collection of the bamboo samples was carried out according to the Convention on Biological Diversity.

## Results and discussion

### Physical properties

Density test was used to measure the density of the samples. The average density of all samples was 594.5 kg/m^3^. The fabricated sample has a low density and according to EN316^36^ standard test method for fiberboard classification, this panel is in the category of light panels and helps to reduce the overall weight of the building significantly compared to the available materials. Obviously, the density factor of the middle layer affects the overall density of the samples, but the other two factors will not affect this value.

### Mechanical properties

Mechanical properties of the panels have been shown in Table 6. According to the EN310 standard test method, the bending strength standard for application in OSB boards with a thickness of 18–25 mm and for general use is equal to 16 MPa. This value is equal to 19.6 MPa for particleboard with a thickness of 25 mm in general use and dry conditions, and equal to 33 MPa for MDF with a thickness of 15 mm. The average modulus of rupture for the samples tested in this study is 19.44 MPa. Considering a model consist of three parameters (A = density, B = core resin and C = face resin), analysis of variance demonstrates a siginificant model (Table 5). The number 4.71 for the F-value indicates the significance of the model, and based on the analaysis of variance only 3.64% error is likely to occur. If the P-value is less than 0.05, the model will be significant. The modulus of rupture can be calculated based on the linear relationship described here (Eq. 1).

1 $$\begin{aligned} & {\begin{aligned} {\text{MOR}} & = { 4}.0{1 } \times {\text{ A }}{-}{ 1}.{24 } \times {\text{ B }}{-}{ 1}.{55 } \times {\text{ C }} + \, 0.{58 } \times {\text{ AB}} \\ & \quad {-} \, 0.{94 } \times {\text{ AC }}{-}{ 2}.{82 } \times {\text{ BC }} + { 5}.{38 } \times {\text{ A2}} \\ & \quad + { 4}.{82 } \times {\text{ B2 }} + { 6}.{63 } \times {\text{ C2}}\end{aligned}} \\ & ({\text{A}}\, = \,{\text{density}},{\text{ B}}\, = \,{\text{core }}\;{\text{resin}},{\text{ C}}\, = \,{\text{face}}\;{\text{ resin}}) \\ \end{aligned}$$

**Table 5:** Average values of the parameters for the panels.

Treatment	Density(kg/m^3^)	Modulus of rupture (MPa)	Modulus of elasticity (MPa)	Internal bonding (MPa)	Compression strength parallel to face grain (MPa)	Compression strength perpendicular to face grain (MPa)	Compression/Shear strength (MPa)	Screw holding (N)
1	656.04 (65.91)	24.06 (4.62)	7069.66 (1410.3)	0.01 (0.01)	15.66 (2.3)	5.56 (0.94)	0.03 (0.04)	913.33 (18.55)
2	620.1 (29.97)	27.72 (8.28)	7823.33 (2163.97)	0.01 (0.01)	20.28 (2.32)	3.65 (0.97)	0.03 (0.04)	946.66 (14.78)
3	669.6 (79.47)	23.65 (4.21)	7663.33 (2003.97)	0.02 (0)	20.01 (2.05)	10.59 (5.97)	0.01 (0.06)	1090 (158.12)
4	543.68 (46.45)	11.69 (7.75)	3512 (2147.36)	0.02 (0)	16.07 (1.89)	1.04 (3.58)	0.01 (0.06)	691.66 (240.22)
5	630.34 (40.21)	31.89 (12.45)	7900 (2240.64)	0.01 (0.01)	13.35 (4.61)	6.97 (2.35)	0.03 (0.04)	903.33 (28.55)
6	535.73 (54.4)	17.16 (2.28)	5668.66 (9.3)	0.02 (0)	17.6 (0.36)	11.34 (6.72)	0.02 (0.05)	1030 (98.12)
7	536.74 (53.39)	15.25 (4.19)	4939.33 (720.03	0.02 (0)	17.26 (0.7)	2.73 (1.89)	0.02 (0.05)	1461.66 (529.78)
8	608.21 (18.8)	9.52 (9.92)	3367 (2292.36)	0.02 (0)	16.42 (1.54)	4.57 (0.05)	0.01 (0.06)	591.66 (340.22)
9	564.85 (25.28)	12.67 (6.77)	2229.5 (3429.86)	0.08 (0.06)	10.06 (7.9)	2.19 (2.43)	0.01 (0.06)	1026.66 (94.78)
10	613.12 (22.99)	31.05 (11.61)	9199 (3539.64)	0.02 (0)	26.26 (8.3)	2.64 (1.98)	0.01 (0.06)	873.33 (58.55)
11	620.9 (30.77)	22.99 (3.55)	7858 (2198.64)	0.07 (0.05)	21.43 (3.47)	3.67 (0.95)	0.06 (0.01)	1303.33 (371.45)
12	546.13 (44)	10.8 (9.26)	2097.33 (3562.03)	0.02 (0)	11.87 (6.09)	1.9 (2.72)	0.09 (0.02)	505 (426.88)
13	608.42 (18.29)	27.69 (8.25)	8431.66 (2772.3	0.06 (0.04)	24.04 (6.08)	5.09 (0.47)	0.53 (0.46)	1266.66 (334.78)
14	602.29 (12.16)	15.33 (4.11)	4576.66 (1082.7)	0.03 (0.01)	21.39 (3.43)	4.47 (0.15)	0.12 (0.05)	598.33 (333.55)
15	561.81 (28.32)	10.17 (9.27)	2555 (3104.36)	0.02 (0)	17.82 (0.14)	2.99 (1.63)	0.18 (0.11)	776.66 (155.22)

**Figure 8:**
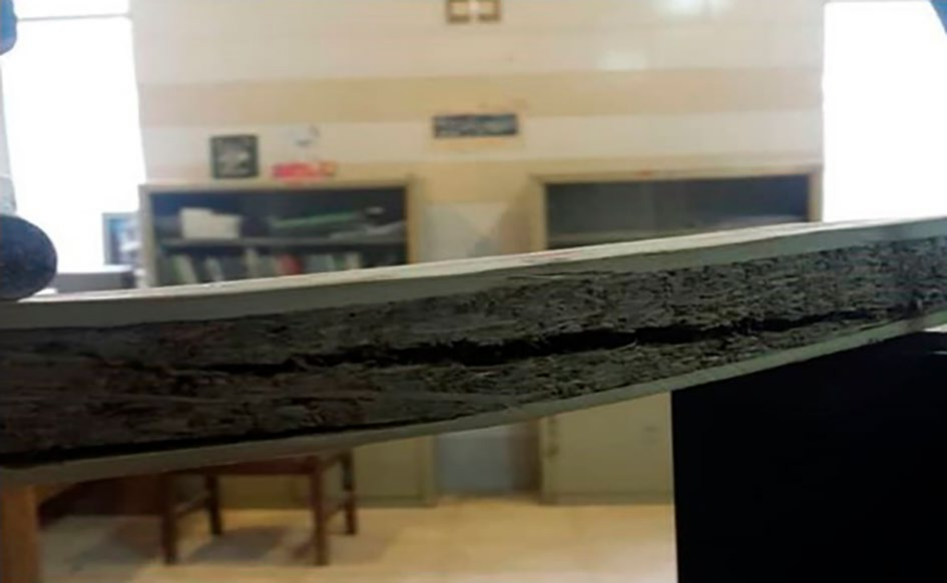
Failure of the core layer due to the compression load.

Considering the construction of the samples, which consist of a particleboard core and two layers of bamboo strips on both sides, it is possible to analyze their impact on bending loads. The middle board breaks due to the applied load (Fig. 8), and by looking at the failure mode, which is diagonal, it can be concluded that the applied compressive force is distributed as a point along the length of the specimen and a shear failure caused the middle board to break. Due to the construction of the core, which is made of bamboo particles impegranted with Polyurethane adhesive, the amount of force applied which caused a fracture in the middle layer is not high, and the modulus of rupture of the specimens can be considered acceptable. The average modulus of elasticity for the constructed specimens is 5659 MPa. As well as MOR, analysis of variance illustrates a significant model for modulus of elasticity. The F-value of 4.53 indicates the significance of the model and only the possibility for an error is 3.98%. The relationship obtained from the model will be significant and the modulus of elasticity can be calculated using (Eq. 2).

2 $$\begin{aligned}& {\begin{aligned} {\text{MOE}} & = { 1273}.{31 } \times {\text{ A }}{-}{ 645}.{46 } \times {\text{ B }} + { 15}.{27 } \times {\text{ C }} + { 185}.{71 } \times {\text{ AB}} \\ & \quad {-}{ 515}.{63 } \times {\text{ AC }}{-}{ 842}.{96 } \times {\text{ BC }} + { 1726}.{8 } \times {\text{ A2 }} \\ & \quad + { 122}0.{86 } \times {\text{ B2 }} + { 1934}.0{1 } \times {\text{ C2}} \end{aligned}} \\ & ({\text{A}}\, = \,{\text{density}},{\text{ B}}\, = \,{\text{core}}\;{\text{ resin}},{\text{ C}}\, = \,{\text{face}}\;{\text{ resin}}) \\ \end{aligned}$$

**Figure 9:**
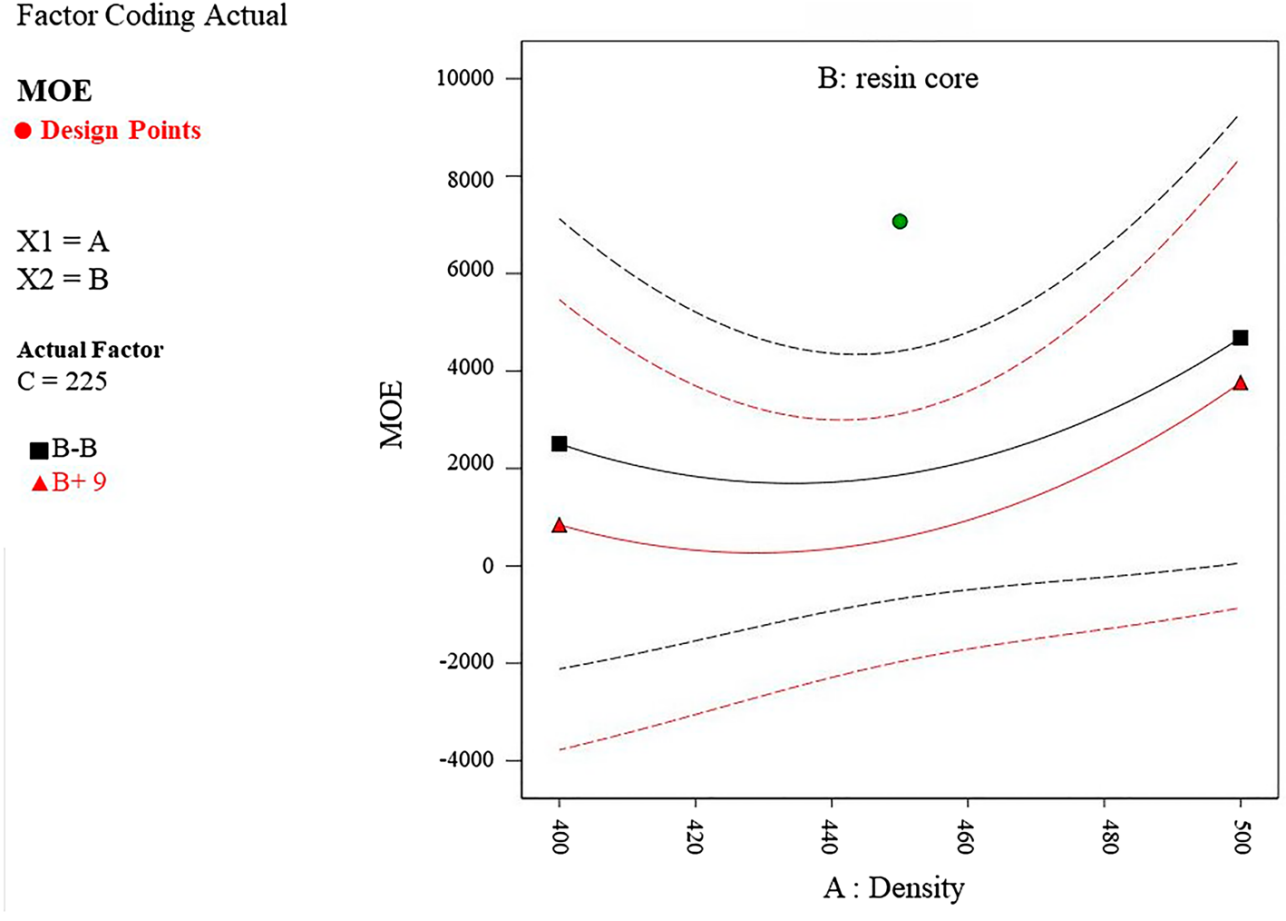
The interaction of density and core resin on MOE.

The core has low flexibility, and due to the strength of the strips on the outer surfaces, the resistance of the boards to deformation is very good, which indicates the appropriate modulus of elasticity of these boards (Fig. 9). The results of internal bonding measurements are between 0.01 and 0.08 MPa, and the average is 0.02 MPa. The number 2.72 indicates the significance of the model and 9.55% is likely to encounter an error. The relationship obtained from the model is not significant.

**Figure 10:**
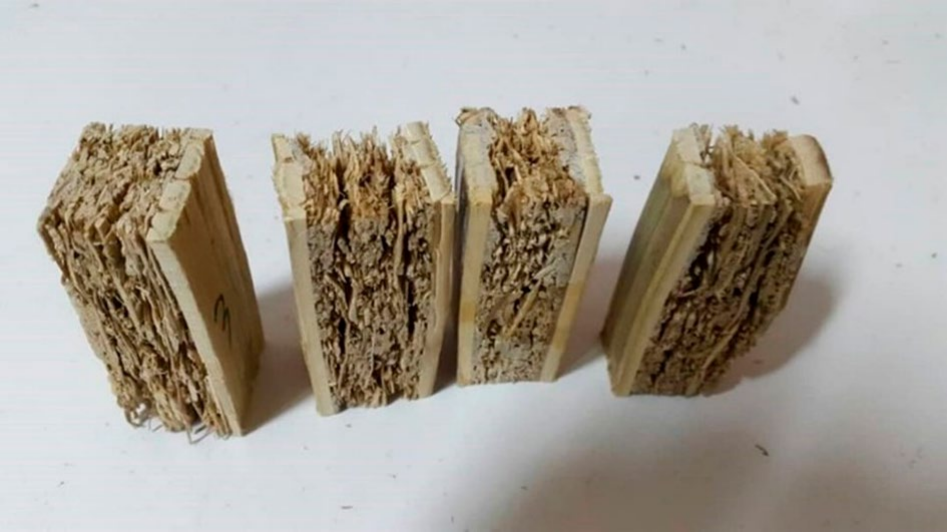
Sample failure modes in compression test parallel to the face grain.

The results of compression strength parallel to face grain measurements showed that this value for the samples is between 10.06 and 26.26 MPa, the average of all the samples tested is 17.6 MPa. The low density of the middle board and its porosity, as well as the amount of adhesive used on the outer surface, have created relatively little adhesion between the strips and the middle board, which disintegrated when the load was applied (Fig. 10). This value will increase as the adhesion improves because the resistance of bamboo strips alone to the parallel compressive force of the fibers is high. This resistance is created due to the structure of bamboo, which is in the form of parallel fibers along the length of its stem. The results of measuring the compression strength perpendicular to face grain showed that this value for the samples is between 1.04 and 11.34 MPa, and the average for all the samples is 4.62 MPa. The analysis of variance in this model shows that it is significant because the F-value is 2.79 and there is only 11.26% possibility for an error (Table 6).

**Table 6:** Analysis of variance of the physical and mechanical properties of the specimens.

Variables sources	Mean square										
	df	MC (%)	Density (kg/m^3^)	TS (%)	MOR (MPa)	MOE (MPa)	IB (MPa)	CSPG (MPa)	CSPPG (MPa)	CS (MPa)	SWR (N)
(A)	1	0.057^n.s^	5902.08^n.s^	56.77^n.s^	257.28^n.s^	2.594E + 07^n.s^	0.0005 ^n.s^	79.3^n.s^	0.41^n.s^	0.04^n.s^	2.332E + 05^n.s^
(B)	1	0.072^n.s^	2062.07^n.s^	61.02^n.s^	24.55^n.s^	6.666E + 06^n.s^	0.0014^n.s^	16.73^n.s^	11.82^n.s^	0.0001^n.s^	74,482.6^n.s^
(C)	1	0.883**	1352.4^n. s^	34.86^n.s^	38.69^n.s^	3731.07^n.s^	0.0011^n.s^	17.72^n.s^	0.9^n.s^	0.0005^n.s^	1501.56^n.s^
(A × B)	1	0.045^n.s^	996.36^n.s^	53.77^n.s^	2.74^n.s^	2.759E + 05^n.s^	0.0000^n.s^	34.78^n.s^	0.31^n.s^	0.004^n.s^	1.031E + 05^n.s^
(A × C)	1	0.003^n.s^	762.06^n.s^	26.04^n.s^	7.14^n.s^	2.127E + 06^n.s^	0.0000^n.s^	3.03^n.s^	10.06^n.s^	0.015^n.s^	52,539.85^n.s^
(B × C)	1	0.026^n.s^	4633.96^n.s^	33.28^n.s^	63.73^n.s^	5.685E + 06^n.s^	0.0000^n.s^	0.51^n.s^	8.97^n.s^	0.005^n.s^	27,415.45^n.s^
A^2^	1	154.23**	9.881E + 05*	1648.82*	1080.66*	1.113E + 08*	0.0009^n.s^	1310.77**	152.09*	0.15*	3.120E + 06**
B^2^	1	133.89**	1.051E + 06*	905.65*	868.31*	5.565E + 07^n.s^	0.0011^n.s^	760.33*	18.01^n.s^	0.0000^n.s^	2.308E + 06*
C^2^	1	117.79*	1.157E + 06*	605.44^n.s^	1643*	1.396E + 08*	0.0009^n.s^	958.84*	62.57^n.s^	0.001^n.s^	2.882E + 06**
Error	6	8.85	86,731.02	144.18	135.35	1.211E + 07	0.0005	736.66	14.37	0.01	1.934E + 05

*Significant at 5% confidence interval, ** Significant at 1% confidence interval, n.s Non-significant; (A) = Density, (B) = Core resin, (C) = Face resin; MC = moisture content, TS = thickness swelling, MOR = modulus of rupture, MOE = modulus of elasticity, IB = internal bonding, CSPG = compression strength parallel to face grain, CSPPG = compression strength perpendicular to face grain, CS = compression-shear strength, SWR = screw withdrawal resistance.

The results of shear test measurements showed that this value for the samples is between 0.01 and 0.532 MPa, the average of all of them is 0.07 MPa (Table 6).

The effect of any of the variable factors on the amount of Compression-shear strength is not significant and based on these variables, the amount of change in this resistance will not be significant (Table 7). The results of measuring the holding force of the screw perpendicular to the surface showed that this value for the samples is between 505 and 1461.70 N, the average of all of them is 931.88 N (Table 6). A value of 7.43 for the F-value indicates the significance of the model and only there are 1.2% chance of possible errors (Table 7). Based on this model, a relationship based on the interaction of factors (density, core resin, face resin) can be defined (Eq. 3) and through it the amount of screw withdrawal resistance (SWR) can be calculated.

3 $$\begin{aligned} & {\begin{aligned} {\text{SWR }} & = { 12}0.{73 } \times {\text{ A }}{-}{68}.{23 } \times {\text{ B }} + { 9}.{69 } \times {\text{ C }}{-}{ 113}.{54 } \times {\text{ AB}} \\ & \quad {-}{ 81}.0{4 } \times {\text{ AC }} + { 58}.{54 } \times {\text{ BC }} + { 289}.0{8 } \times {\text{ A2 }} \\ & \quad + { 248}.{66 } \times {\text{ B2 }} + { 277}.{83 } \times {\text{ C2}} \end{aligned} } \\ & ({\text{A}}\, = \,{\text{density}},{\text{ B}}\, = \,{\text{core}}\;{\text{ resin}},{\text{ C}}\, = \,{\text{face }}\;{\text{resin}}) \\ \end{aligned}$$

**Table 7:** Specific characteristics of different wood-based materials.

NO.	Material	Density (kg/m^3^)	MOR (MPa)	MOE (MPa)	References
1	Light thick Bamboo sandwich panel (LTBSP)	594.5	19.44	5659	The present work
2	Laminated compressed composite panels from oil palm fronds using PF	680	47.35	4355	^45^
3	Hybrid plywood from oil palm biomass using PF	760	50	5012	^46^
4	Date palm biomass particleboards using PF	700	26	4243	^47^
5	Eastern red cedar sandwich panel using PF	600	27.5	4417	^48^
6	MF/UF bonded Chipboard	660	41	5242	^31^
7	Lightweight Date palm wood–MDF sandwich panel	456	67.08	6707.79	^31^

**Figure 11:**
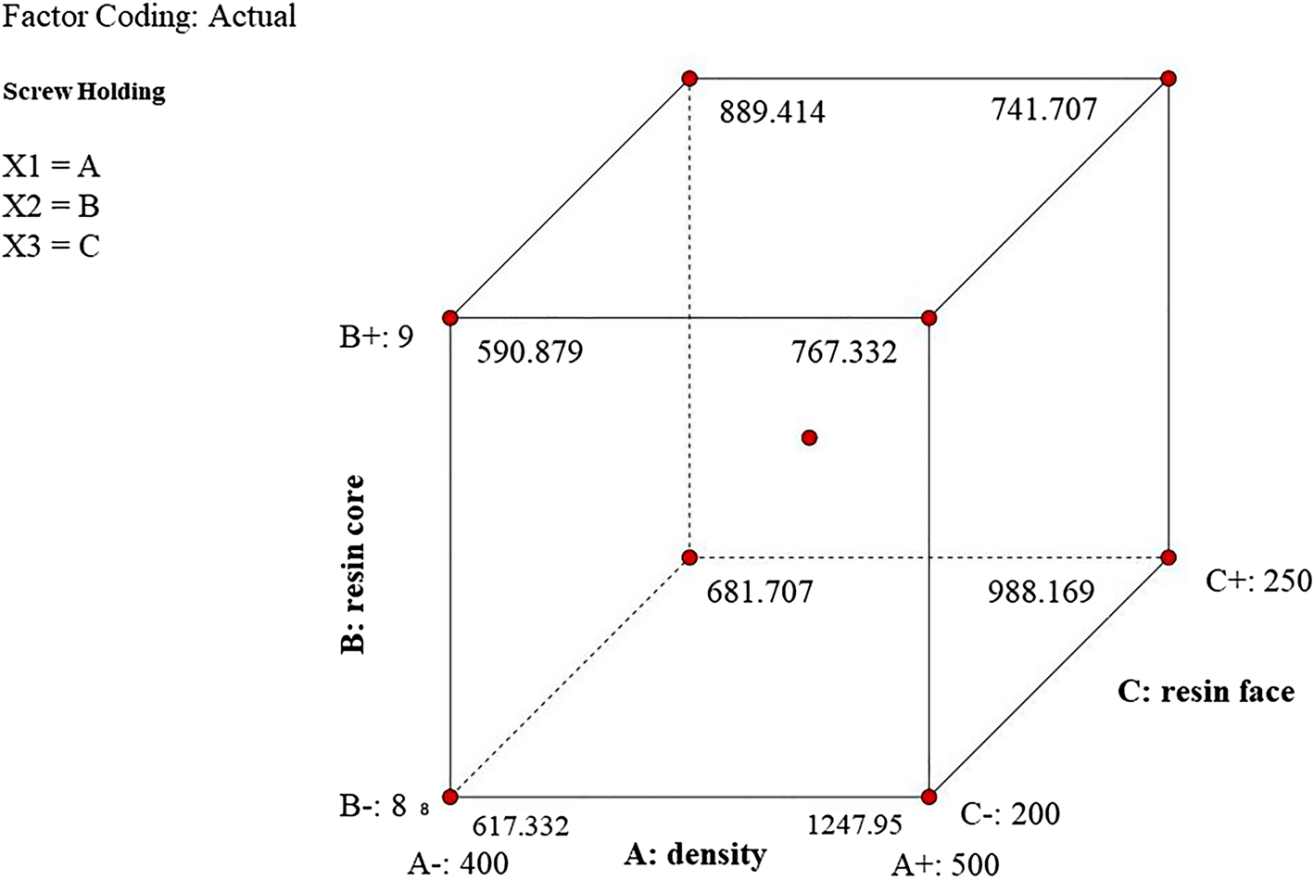
The interaction of density, core resin and face resin on SWR.

The high density and strength of bamboo strips on external surfaces increases the amount of screw withdrawal resistance in the fabricated sample (Fig. 11). The use of bamboo strips in the construction of the samples has made the modulus of elasticity of the product fall into the right category compared to some other products, such as Laminated compressed composite panel from palm fronds, Hybrid plywood from oil palm biomass and Date palm biomass particleboards (Table 7). Due to lower density of this composite and its production method, which includes a core layer made of bamboo particleboard, the modulus of rupture is weaker than that of other products and needs to be improved (Table 7).

### Thermo-acoustic performance

Test specimen with code 13 (Table 5) has the highest density among the samples, which is equal to 669.6 kg/m^3^. To calculate the noise reduction coefficient for a sample, the average sound absorption coefficient at frequencies of 250, 500, 1000 and 2000 must be taken. For sample 13 the absorption coefficient was 0.3125. Test sample with code 6 has the lowest density among the samples, which is 536.74 kg/m^3^. Due to the low density, it was not possible to cut a disc with a diameter of 29 mm for this test and therefore it was measured only at low frequencies. The average value for this test was obtained from the frequencies of 250, 500 and 1000 which is equal to 0.4633. The average density of the samples is 594.5 kg/m^3^ and the average thermal conductivity is 0.0199 W/mK. The results of the tests indicate that the product is desirable in terms of sound absorption and thermal conductivity (Tables 8, 9). The average MOR and MOE values obtained from bamboo panels are comparable to other materials as shown in Table 8. High percentage of porosity and low density of boards are effective in increasing the coefficients and comparison of samples with similar products shows the effect of the middle board on both coefficients. It should be noted that the presence of bamboo strips on the two outer surfaces of the boards has an effect on reducing the sound absorption coefficient and has caused the sound to be reflected from the surface of the sample. Due to the use of bamboo and fabrication of low density samples, the thermal conductivity is also very desirable according to the test results (Table 9). These new structures are important in several ways due to their lightness and high insulation properties. The low thickness of this composite (2.5 cm) and at the same time its multiple function is the first point. In addition to its separating function and beauty, this structure is also a very good insulator. Another point is that these structures are more sustainable than other structures. If common materials are used, add-ons should be used on the wall to insulate it, however, these panels are also insulated by themselves. This saves energy and is more sustainable.

**Table 8:** Noise reduction coefficient for different materials^24^.

Material	Density (kg/m^3^)	NRC
Light thick Bamboo sandwich panel (LTBSP)	594.5	0.3125
Wadding material	20	0.31
Wood wool building slab	–	0.3
Bagasse particleboard with MUF resin	190–460	0.215–0.38
Wood	–	0.05–0.15
Plywood	–	0.1–0.15
Brick (unpainted)	–	0.00–0.05
Concrete (smooth-unpainted)	–	0.00–0.2
Concrete (block-unpainted)	–	0.05–0.35
Gypsum	–	0.05
Fiberglass (semi rigid)	–	0.5–0.75

**Table 9:** Thermal conductivity coefficient for different materials.

Material	Thermal conductivity (W/mK)	References
Light thick Bamboo sandwich panel (LTBSP)	0.0199	This job
Wood (pine, lauan)	0.151	^49^
Brick	0.62	^50^
Plywood (beech)	0.1304	^51^
Eastern red cedar	0.14	^52^
Gypsum fibreboard	0.32	^53^

### Optimization

To select the optimal treatment, the Design Expert software optimization plugin was used. The treatment was selected based on the significant results of the tests to increase the desirability. In the analysis for optimizing, only the significant results were considered, these tests are: modulus of rupture, modulus of elasticity, compressive strength perpendicular to the face grains and screw withdrawal strength. Variable factors that were: middle layer density, core layer resin and outer surface adhesive were considered in their specific range. The selected treatment has a desirability of 1. Tables 10 and 11 show the complete specifications of the optimal treatment.

**Table 10:** Optimal treatment conditions.

Name	Goal	Lower limit	Upper limit	Lower weight	Upper weight	Importance
A: density	Is in range	350	550	1	1	3
B: resin core	Is in range	7.5	9.5	1	1	3
C: resin face	Is in range	175	275	1	1	3
IB	None	0.01	0.08	1	1	3
MOR	Maximize	9.52	31.89	1	1	5
MOE	Maximize	2097.33	9199	1	1	5
Compression parallel to grain	None	10.06	26.26	1	1	3
Compression perpendicular to grain	None	1.04	11.34	1	1	3
Shear	None	0.01	0.53	1	1	3
Screw holding	Maximize	505	1461.66	1	1	3
Density	None	535.73	669.6	1	1	3
H	None	5.62	7.52	1	1	3

**Table 11:** Optimal treatment specifications.

Density of core layer	Core resin	Face resin	IB	MOR	MOE	CPaG	CPeG	SHS	SWR	Density	H	Desirability
464.462	7.515	176.489	0.043	40.176	10,737.447	35.906	4.361	0.129	2520.224	1234.185	14.299	1

IB, internal bonding; MOR, modulus of rupture; MOE, modulus of elasticity; CPaG, compression parallel to grain; CPeG, compression perpendicular to grain; SHS, shear strength; SWR, screw withdrawal resistance; H, humidity.

## Conclusion

To conclude, in this paper a series of physical, mechanical and insulating performance of a novel light bamboo sandwich panel (LTBSP) as a sustainable green composite (manily based on waste and renewable resources) from bamboo strips and shavings was investigated.

- LTBSP stood a better chance to be applied as a wall panel- according to test results that mentioned before- rather than MDF or knauf wall boards.
- Some of the mechanical properties of LTBSP such as modulus of rupture, modulus of elasticity and resistance to axial withdrawal of screws reached the requirements of DIN-EN 312–320 standard test methods.
- The thermal conductivity and noise reduction coefficients for LTBSP met the requirements of IS3129-1985 for particleboards.
- In view of test results obtained in this study and in comparison to other composites, LTBSP is a suitable material as a wall panel, considering its components that are environment-friendly, it can be seen as an alternative to synthetic-based commercial products.

## Data Availability

The data that support the fndings of this study are available from the corresponding author, AJ, upon reasonable request.
